# Rosuvastatin-Induced Dizziness and Pruritus: A Case Report and Summary of Statin-Associated Dizziness and Pruritus

**DOI:** 10.7759/cureus.29014

**Published:** 2022-09-10

**Authors:** Sara Malik, Philip R Cohen

**Affiliations:** 1 Feinberg School of Medicine, Northwestern University, Chicago, USA; 2 Dermatology, University of California, Davis Medical Center, Sacramento, USA

**Keywords:** triglycerides, statin, side, rosuvastatin, pruritus, medication, hyperlipidemia, effect, dizziness, adverse

## Abstract

Drug-associated adverse events can present with varying symptoms, such as dizziness and pruritus. A 48-year-old woman initiated rosuvastatin to treat her elevated triglycerides. She developed rosuvastatin-associated adverse events, which included dizziness and pruritus within two weeks after starting treatment. After stopping the medication, the dizziness immediately cleared; the pruritus diminished during the next two weeks and eventually resolved completely. Side effects associated with rosuvastatin are discussed. The possibility of a medication-related etiology should be entertained when an individual suddenly develops either dizziness or pruritus or both.

## Introduction

Dizziness describes a range of sensations that can vary from feeling faint and lightheaded to weak and unsteady. In contrast, vitiligo is a false sense that a person’s environment is spinning. Eliciting a comprehensive history and performing a complete physical examination can aid in determining the cause of dizziness. One possible cause of dizziness is an adverse effect of medication; therefore, the patient’s drug regimen should be assessed if they present with dizziness [1].

Pruritus is an unpleasant feeling that causes the desire to scratch. Etiologies of itching include not only internal factors, such as infection, endocrine disorders, and metabolic diseases, but also external factors such as food, inhaled substances, and contact of the skin with irritants or allergens. Common causes of pruritus include contact dermatitis, eczema, and urticaria. Medications can also cause pruritus; thus, a complete history and laboratory studies may be helpful to determine the etiology of pruritus [2].

Statins are a class of drugs that inhibit the hydroxymethylglutaryl-CoA (HMG-CoA) reductase enzyme. Statins are prescribed for the prevention of cardiovascular disease because they are lipid-lowering agents; particularly, they are effective in decreasing low-density lipoprotein cholesterol in the blood. Statin-associated adverse events include hepatotoxicity, myalgias, and other muscle-related side effects such as weakness [3].

A woman with hyperlipidemia, characterized by an elevated triglyceride level and a normal cholesterol level, was evaluated by her primary care physician who decided to begin treatment with rosuvastatin. Within two weeks, she developed dizziness and pruritus. Both symptoms resolved after discontinuing the medication. Drug-related side effects of rosuvastatin are discussed, and the occurrence of statin-associated dizziness and pruritus are summarized.

## Case presentation

A 48-year-old woman was referred to a dermatologist for generalized pruritus. Her past medical history was significant for hyperlipidemia, mastodynia, obesity, and viral warts. Prior drug allergies included penicillin, morphine, and sulfamethoxazole/trimethoprim. In addition to recently beginning rosuvastatin, her chronic medications included diclofenac sodium, fluticasone, gabapentin, glucosamine chondroitin, ibuprofen, lubiprostone, and omeprazole. 

Additional history revealed that she not only developed pruritus but also concurrently experienced lightheadedness and feeling faint; on several occasions, she mentioned her episodes of dizziness to her family members. Both symptoms began within two weeks after starting rosuvastatin. She independently decided to discontinue the new medication. Less than one day later, the dizziness resolved; the pruritus gradually improved by the time she was evaluated by a dermatologist. 

A complete cutaneous examination was performed. There were resolving excoriations on her back and arms. There was neither evidence of burrows (which would have been suggestive of scabies) nor any lesion associated with an itch-related primary dermatosis (such as dermatitis, lichen planus, or psoriasis). 

The correlation of the patient’s history and clinical presentation, including the resolution of her symptoms after discontinuing the newly started medication, established a diagnosis of rosuvastatin-induced dizziness and pruritus. Since her symptoms had already improved significantly, daily topical application of an emollient cream was recommended to moisturize her skin. Subsequently, the pruritus completely resolved during the next four weeks. 

The patient returned to her primary care physician. They discussed restarting rosuvastatin to confirm the occurrence of dizziness and pruritus as an adverse reaction to the drug; however, she declined this option. She began treatment with gemfibrozil without any adverse systemic or cutaneous reactions. 

## Discussion

Rosuvastatin is a lipid-lowering drug that belongs to the statin class of medications. It is used to treat patients with increased cholesterol and triglycerides. Dosing of rosuvastatin is based on a patient’s baseline lipid profile; daily doses range from 5 to 40 milligrams. Adverse effects of rosuvastatin include elevated creatinine kinase levels, elevated liver enzymes, myopathy, renal failure, and rhabdomyolysis [4]. 

Rosuvastatin-induced dizziness has previously been observed (Table 1) [3,5-10]. In a study of 12 patients who were started on rosuvastatin, two patients developed dizziness [5]. In another study of 172 patients, two individuals experienced dizziness [6]. Furthermore, in a study of 36 participants that was investigating the effects of coadministration of rosuvastatin and olmesartan, dizziness occurred in one participant who received only rosuvastatin in addition to four participants who received both rosuvastatin and olmesartan, further suggesting that rosuvastatin can elicit dizziness [7]. In another study in which 36 patients received either fimasartan and/or rosuvastatin, one individual experienced dizziness [8]. In a research study of 375 patients, two individuals experienced dizziness: one who received rosuvastatin and one who received rosuvastatin and ezetimibe [9]. The investigators of a final study, which included 42 patients who received rosuvastatin, observed one individual with dizziness [10]. 

**Table 1:** Incidence of rosuvastatin-induced dizziness ^a^Pharmacokinetic studies have suggested that patients of East Asian ethnicity, compared to Caucasians, have greater plasma concentrations of some statins and their active metabolites. Therefore, recommendations for prescribing lower doses of statins have been considered for East Asians who have an increased risk of developing adverse effects (particularly myopathy and rhabdomyolysis) at higher doses [3]. ^b^Two women, receiving 20 milligrams daily, experienced dizziness and simultaneous cold sweats. They were successfully treated with oral glucose. ^c^Dizziness and edema, each with an incidence of 1.2 percent, were the most frequent adverse events in the patients receiving rosuvastatin. ^d^Of the participants who received rosuvastatin and had dizziness, one participant only received rosuvastatin; therefore, dizziness was solely attributed to rosuvastatin in one patient. However, dizziness was also observed in patients receiving olmesartan either alone (one patient) or associated with rosuvastatin (four patients). ^e^Postural dizziness was attributed to both rosuvastatin and fimasartan. ^f^Dizziness occurred in one patient receiving 20 milligrams rosuvastatin daily and one patient receiving both 20 milligrams rosuvastatin and 10 milligrams ezetimibe daily. ^g^Dizziness was observed in one patient receiving 20 milligrams rosuvastatin daily. Dizziness was also observed in three patients receiving 80 milligrams telmisartan and one patient receiving placebo (no drug).

Author^a^	Year	Number of patients	Number of patients with dizziness	Percentage of patients with dizziness	References
Zhang et al.^b^	2009	12	2	16.7	[5]
Park et al.^c^	2010	172	2	1.2	[6]
Roh et al.^d^	2014	36	5	13.9	[7]
Lee et al.^e^	2018	36	1	2.8	[8]
Kim et al.^f^	2018	375	2	0.5	[9]
Oh et al.^g^	2018	42	1	2.4	[10]

An estimate of the occurrence of rosuvastatin-induced dizziness, by combining the data from the studies in Table 1 that included patients from Southeast Asian countries, shows an incidence of 1.9 percent (13 of 673 patients) [3,5-10]. The reported patient developed dizziness within two weeks of starting rosuvastatin. Her dizziness resolved almost immediately after discontinuation of the medication. 

Rosuvastatin-induced pruritus has also been noted in patients treated with the drug. A 30-year-old-man with a history of hyperlipidemia and myocardial infarction developed an anaphylactic reaction, which included pruritus, to both atorvastatin and simvastatin independently. Upon the initiation of desensitization with rosuvastatin, the man also developed pruritus [11]. Similarly, a 29-year-old woman with familial hypercholesterolemia developed an allergic reaction to atorvastatin and simvastatin. After starting rosuvastatin for desensitization, she experienced pruritus and flushing [12]. Subsequently, although both patients initially experienced rosuvastatin-associated pruritus, they were able to undergo successful desensitization to the drug [11,12]. 

Recent studies of patients from Southeast Asian countries who were receiving rosuvastatin showed a low incidence of drug-associated pruritus. Indeed, the observed occurrence of rosuvastatin-related pruritus ranged from 0.2 percent to 1.2 percent (Table 2) [3,9,10]. Combining the data from the studies in Table 2 shows an incidence of 0.4 percent (two of 457 patients) [3,9,10].

**Table 2:** Incidence of rosuvastatin-induced pruritus ^a^Pharmacokinetic studies have suggested that patients of East Asian ethnicity, compared to Caucasians, have greater plasma concentrations of some statins and their active metabolites. Therefore, recommendations for prescribing lower doses of statins have been considered for East Asians who have an increased risk of developing adverse effects (particularly myopathy and rhabdomyolysis) at higher doses [3]. ^b^Pruritus was observed in a patient on both 20 milligrams rosuvastatin and 10 milligrams ezetimibe. ^c^Pruritus was observed in a patient on combination therapy of 20 milligrams rosuvastatin and 80 milligrams telmisartan.

Author^a^	Year	Number of patients	Number of patients with pruritus	Percentage of patients with pruritus	References
Kim et al.^b^	2018	375	1	0.2	[9]
Oh et al.^c^	2018	82	1	1.2	[10]

Statin-associated dizziness or pruritus is not limited to rosuvastatin. Dizziness and pruritus have also independently been observed in patients taking other statins including atorvastatin, fluvastatin, lovastatin, pitavastatin, pravastatin, and simvastatin (Table 3) [5,7-10,13-19]. However, there are no studies that evaluated whether high-intensity statins are more likely to cause dizziness and/or pruritus as compared to low-intensity statins.

**Table 3:** Observation of statin-associated dizziness or pruritus Abbreviation: + indicates symptom has been described ^a^In a study of 1690 participants receiving either atorvastatin, fluvastatin, pitavastatin, pravastatin, rosuvastatin, or simvastatin, 103 (4.8 percent) of the patients experienced pruritus and 94 (4.4 percent) of the patients experienced dizziness. ^b^One of 35 patients receiving 20 milligrams fluvastatin daily developed dizziness (in addition to blurred vision and nausea) and discontinued the study. ^c^Dizziness was observed in 317 of 3,304 (ten percent) of patients receiving lovastatin. ^d^A 40-year-old man presented with severe itching 11 weeks after starting lovastatin secondary to drug-induces cholestatic jaundice. ^e^One of 42 patients receiving 20 milligrams pravastatin daily withdrew from the study because of dizziness. ^f^Simvastatin was stopped in one of 24 patients who developed not only dizziness but also abdominal pain, headache, and tiredness. ^g^A 67-year-old patient presented with pruritus and jaundice, secondary to acute cholestatic hepatitis, after initiating treatment with simvastatin.

Statin	Dizziness	Pruritus	References
Atorvastatin^a^	+	+	[13]
Fluvastatin^a,b^	+	+	[13,14]
Lovastatin^c,d^	+	+	[15,16]
Pitavastatin^a^	+	+	[13]
Pravastatin^a,e^	+	+	[13,17]
Rosuvastatin^a^	+	+	[5,7-10,13]
Simvastatin^a,f,g^	+	+	[13,18,19]

The reported patient was offered to restart rosuvastatin to establish unequivocally that her symptoms were drug-associated, but she declined to take the medication. She started a new drug for her hyperlipidemia, gemfibrozil, and did not experience any reactions to the drug. According to the Naranjo scale of adverse drug reaction probability, both symptoms received a score of eight, which implies that the dizziness and pruritus she experienced were probable reactions to rosuvastatin (Table 4) [5-10,20].

**Table 4:** Patient’s Naranjo scale score for dizziness and pruritus Abbreviations: +, add; -, subtract ^a^There are published reports of either dizziness or pruritus secondary to rosuvastatin [5-10] ^b^After rosuvastatin was discontinued, the patient received a non-statin triglyceride-lowering drug: gemfibrozil. Since the new medication was anticipated to have a therapeutic effect, it is not a true placebo; however, therapy with the new fibrate agent (gemfibrozil) may be considered a placebo-like treatment in comparison to the statin class of drug (rosuvastatin) that she was previously receiving. ^c^The patient informed her family about the episodes of lightheadedness and feeling faint that she was experiencing. Resolving excoriations, and evidence of her pruritus were observed during her cutaneous examination. ^d^Naranjo scale scores are as follows: a score of greater than or equal to nine means that there is a “definite” probability that the adverse event was related to the drug, a score of five to eight means that it was a “probable” reaction, a score of one to four means that it was a “possible” reaction, and a score of zero or below means that the reaction was defined as “doubtful.” Our patient’s score of eight means that her dizziness and pruritus was a “probable” reaction to rosuvastatin. The reaction followed a reasonable temporal sequence after the administration of rosuvastatin, had a recognized response to rosuvastatin, was confirmed by withdrawal—but not repeat exposure to—rosuvastatin, and could not be reasonably explained by the known characteristics of the patient’s clinical state [20].

Question	Yes	No	Do not know	Patient score
1. Are there previous conclusive reports on this reaction?	+1	0	0	+1^a^
2. Did the adverse event appear after the suspected drug was administered?	+2	-1	0	+2
3. Did the adverse event improve when the drug was discontinued or a specific antagonist was administered?	+1	0	0	+1
4. Did the adverse event reappear when the drug was readministered?	+2	-1	0	0
5. Are there alternative causes that could on their own have caused the reaction?	-1	+2	0	+2
6. Did the reaction reappear when a placebo was given?	-1	+1	0	+1^b^
7. Was the drug detected in blood or other fluids in concentrations known to be toxic?	+1	0	0	0
8. Was the reaction more severe when the dose was increased or less severe when the dose was decreased?	+1	0	0	0
9. Did the patient have a similar reaction to the same or similar drugs in any previous exposure?	+1	0	0	0
10. Was the adverse event confirmed by any objective evidence?	+1	0	0	+1^c^
	Total Score: 8^d^			

There are no previous case reports of concurrent dizziness and pruritus associated with rosuvastatin. However, it is important for clinicians to recognize that new symptoms can be a result of an adverse effect of a medication. The initial treatment of medication-associated dizziness and pruritus is the discontinuation of the drug. Usually, both symptoms will spontaneously resolve once the causative agent has been stopped. 

## Conclusions

Rosuvastatin has been associated with several adverse events. Albeit less common, dizziness and pruritus can occur following initiation of treatment with rosuvastatin. A 48-year-old woman developed not only dizziness but also pruritus within two weeks of starting rosuvastatin. After the medication was discontinued, her dizziness resolved immediately and her pruritus significantly improved within two weeks. To the best of our knowledge, concurrent rosuvastatin-induced dizziness and pruritus have only been described in the reported woman. In addition to rosuvastatin, dizziness and pruritus have occurred in patients treated with other statins. Therefore, clinicians should consider the possibility of medication-associated etiology when a patient presents with a new onset of dizziness of generalized pruritus, or both.
